# Supplementary material for: Influence of agriculture on child nutrition through child feeding practices in India: A district-level analysis
Source: PLoS One. 2021 Dec 16;16(12):e0261237. doi: 10.1371/journal.pone.0261237 (PMC8675687; doi:10.1371/journal.pone.0261237)
Supplement: Attachment — Submitted filename: PONE-D-21-18510 Review.docx [file pone.0261237.s001.docx]

Review notes on

Influence of Agriculture on Child Nutrition through Child Feeding Practices in India: A District-Level Analysis (PONE-D-21-18510)

(Research Article)

Manas Ranjan Pradhan (Corresponding Author)

International Institute for Population Sciences, India

In this current research work, the authors have nicely presented influence of agriculture on child nutrition in India. However, there can be some improvements in the current manuscript. They are as follows-

1. Authors must add a conceptual or theoretical graphical framework in the introduction part of the present paper.
2. Materials and method section can be modified by defining the study districts, state, and the country more prominently under the study area section.
3. In analysis part, for deeper understanding and to find out stronger predictor variables the authors can go for stepwise regression, path, and principal component analysis (PCA). Additionally, authors must go for data visualization after the analyses.
4. In this present study, the total calorie consumed by the child per day in the study districts of India, can be added as an important independent variable.
5. BMI of both the mother and child can also be considered as one important dependent variable.
6. Some more references must be added in this current study.
