# Supplementary material for: Influence of agriculture on child nutrition through child feeding practices in India: A district-level analysis
Source: PLoS One. 2021 Dec 16;16(12):e0261237. doi: 10.1371/journal.pone.0261237 (PMC8675687; doi:10.1371/journal.pone.0261237)
Supplement: Attachment — Submitted filename: R1_Response to reviewer_070921.docx [file pone.0261237.s002.docx]

Review notes on

Influence of Agriculture on Child Nutrition through Child Feeding Practices in India: A District-Level Analysis (PONE-D-21-18510)

(Research Article)

Manas Ranjan Pradhan (Corresponding Author)

International Institute for Population Sciences, India

**Journal Requirements:**

Comment 1: Please ensure that your manuscript meets PLOS ONE's style requirements, including those for file naming.

**Response 1**: **We have checked the PLOS ONE's style requirements, including those for file naming.**

Comment 2. Please address the following:

- Please refrain from stating p values as 0.000 and instead use the format p<0.0001.

- Please ensure you do not have any statements of causation within your Conclusions, as these cannot be confirmed following a study of this type

**Response 2: We have revised the manuscript as suggested**

**Additional Editor Comments (if provided):**

**Comment**: While the manuscript has contained good information, the needs a better organization, editing and data analysis and interpretation for addressing food availability and quality related to children health. Also, address abbreviations and acronyms and in the abstract with data presentation in the table and figures with significant digits. There were 15 graphs which could be combined at 4 in 1. Important regression analyses can be shown in figures for better depiction of the results and interpretation.

**Response**: **We have revised the paper in view of the comments. Specifically, addressed abbreviations and acronyms in the abstract, data presentation in the tables with significant digits, combined the graphs and presented the important regression results in figure**.

**Reviewer Comments**

In this current research work, the authors have nicely presented influence of agriculture on child nutrition in India. However, there can be some improvements in the current manuscript. They are as follows-

**Response: Thank you for reviewing our article and for the critical comments to improve the quality of the article. We have revised the article in view of the comments.**

Comment 1: Authors must add a conceptual or theoretical graphical framework in the introduction part of the present paper.

**Response 1: We have added a conceptual framework as per the suggestion.**

Comment 2: Materials and method section can be modified by defining the study districts, state, and the country more prominently under the study area section.

**Response 2: We have revised the materials and method section as suggested. Specifically, we have added a section on study area.**

Comment 3: In analysis part, for deeper understanding and to find out stronger predictor variables the authors can go for stepwise regression, path, and principal component analysis (PCA). Additionally, authors must go for data visualization after the analyses.

**Response 3: The authors have done data visualization as suggested. Specifically, the OLS regression result for Minimum Acceptable Diet (MAD) is presented through a forest plot. We restricted ourselves from making more such figures to limit the number of figures. The stepwise regression, path and PCA are alternative ways to look into the data though they do have disadvantages as well. We think OLS analysis is equally appropriate, given the objectives of the study and the nature of the data considered for this analysis.**

Comment 4: In this present study, the total calorie consumed by the child per day in the study districts of India, can be added as an important independent variable.

**Response 4: Thank you for the suggestion. However, the survey data considered for this analysis does not have this information. Nevertheless, we believe that MAD which is a composite indicator composed of MMF and MDD, indicate household calorie availability as well as consumption and hence can be considered as a proxy for calorie intake.**

Comment 5: BMI of both the mother and child can also be considered as one important dependent variable.

**Response 5: In this paper the authors aim is to assess the influence of agriculture on child undernutrition measured through stunting, wasting and underweight. To make the analysis focused, the authors did not include other associated outcome variables. Moreover, we have used the BMI of the mother as predictor variable in the analysis.**

Comment 6: Some more references must be added in this current study.

**Response 6: We have added additional references in the revised manuscript**
